# Beyond Cardiorenal Risk Factors: The Prognostic Role of Frailty in Acute Heart Failure

**DOI:** 10.3390/medicina62101942

**Published:** 2026-10-08

**Authors:** Georgios Aletras, Maria Marketou, Maria Bachlitzanaki, Maria Stratinaki, Maria Tsigaridaki, Yannis Pantazis, Emmanuel Foukarakis, Michalis Hamilos, Konstantinos Stylianou

**Affiliations:** 1 Department of Cardiology, Venizelio General Hospital of Heraklion, 71409 Heraklion, Greece; aletras.george@gmail.com or medp2012222@med.uoc.gr (G.A.); maria.stratinaki@gmail.com (M.S.); mfouk@hotmail.com (E.F.); 2 School of Medicine, University of Crete, 70013 Heraklion, Greece; mchamilos@uoc.gr (M.H.); kstylianu@med.uoc.gr (K.S.); 3 Department of Cardiology, University General Hospital of Heraklion, 71500 Heraklion, Greece; 4 Second Department of Internal Medicine, Venizelio General Hospital of Heraklion, 71409 Heraklion, Greece; mariabachlitzanaki@gmail.com; 5 Department of Biochemistry, Venizelio General Hospital of Heraklion, 71409 Heraklion, Greece; tsigaridaki.m@gmail.com; 6 Institute of Applied and Computational Mathematics, Foundation for Research and Technology–Hellas, 70013 Heraklion, Greece; pantazis@iacm.forth.gr; 7 Department of Nephrology, University General Hospital of Heraklion, 71500 Heraklion, Greece

**Keywords:** acute heart failure, frailty, Clinical Frailty Scale, cardiorenal factors, prognosis, risk stratification

## Abstract

***Background:*** Frailty is highly prevalent in acute heart failure (AHF), yet whether it is more strongly associated with all-cause mortality or with non-fatal heart-failure events, and whether it adds information beyond cardiac severity, is seldom examined. ***Methods:*** In a prospective single-center registry of 530 consecutive patients hospitalized for AHF (February 2023–June 2025), frailty was graded at admission by the Clinical Frailty Scale (CFS; strata 1–3, 4–5, 6–9, and CFS ≥ 5) from patient and collateral history. The primary outcome was 12-month all-cause mortality; first AHF rehospitalization was analyzed with death and renal replacement therapy (RRT) as competing events, using Aalen–Johansen cumulative incidence and cause-specific and Fine–Gray models. Incremental value was tested against a cardiorenal model (age, log NT-proBNP, admission estimated glomerular filtration rate and left ventricular ejection fraction [LVEF]). ***Results:*** Frailty was highly prevalent (CFS ≥ 5, 66.4%) and was associated with older age, female sex, renal impairment, right heart failure, multivalvular disease and higher, rather than lower, LVEF. Mortality rose steeply across strata (4.5%, 19.3%, 49.7%; hazard ratio 3.29 per stratum, 95% CI 2.43–4.45), whereas the cumulative incidence of first AHF rehospitalization rose less steeply (4.5%, 29.2%, 33.0%; cause-specific hazard ratio 1.86, 95% CI 1.42–2.45; subdistribution hazard ratio 1.51, 95% CI 1.19–1.92). Frail patients with below-median NT-proBNP had mortality comparable to that of patients with CFS < 5 and above-median NT-proBNP (24.3% vs. 22.0%). Adding CFS to the cardiorenal model improved discrimination (C-index 0.701 → 0.734; optimism-corrected 0.694 → 0.726; apparent ΔC-index 0.033, 95% CI 0.007–0.063) and fit (likelihood-ratio χ^2^ = 27.7, *p* < 0.001). Discharge prescription of disease-modifying therapy declined with increasing frailty. ***Conclusions:*** In elderly patients with AHF, frailty was more strongly associated with all-cause mortality than with documented non-fatal AHF rehospitalization, carried prognostic information complementary to natriuretic peptides, and added discrimination beyond cardiorenal variables, supporting structured frailty assessment alongside heart-failure-directed therapy.

## 1. Introduction

Acute heart failure (AHF) is a leading cause of hospitalization and death in older adults [1,2], and contemporary patients presenting with AHF are increasingly older and more multimorbid than those enrolled in landmark trials and registries. In this context, frailty—a state of diminished physiological reserve and heightened vulnerability to stressors—has emerged as a central, yet still under-recognized, determinant of outcome. Frailty is present in one- to two-thirds of hospitalized HF patients depending on the instrument used [3,4,5], and consistently identifies patients at higher risk of death and disability [6,7].

The Clinical Frailty Scale (CFS) is a simple, judgement-based, nine-level instrument that integrates comorbidity, function and cognition, and is readily applied at the bedside [8,9]. Although frailty is repeatedly reported to predict mortality in HF [10,11], most analyses treat it as one covariate among many in a mortality model, leaving unresolved a more fundamental question: the nature of the prognostic signal conveyed by frailty remains uncertain. Specifically, it is unclear whether frailty primarily increases the risk of HF-related events or instead reflects vulnerability to competing non-cardiac mortality and functional decline that cannot be addressed by intensification of HF therapy alone.

We therefore approached frailty not as a confounder but as the exposure of interest. In a prospective AHF registry with complete one-year follow-up, we (i) characterized the clinical, laboratory and echocardiographic phenotype associated with increasing frailty; (ii) analyzed its outcome profile cause-specifically, separating all-cause death from AHF-specific rehospitalization under a competing-risks framework; (iii) tested whether frailty is concordant or discordant with natriuretic-peptide elevation; and (iv) quantified the incremental discrimination that frailty adds to a cardiorenal reference model. Because prior studies, including large registries, have already reported associations of the CFS with both death and HF rehospitalization, the intended contribution of this work is not the general prognostic observation but the competing-risks separation of all-cause mortality from non-fatal events and the assessment of incremental value beyond established cardiorenal variables.

## 2. Materials and Methods

### 2.1. Study Design and Population

**Figure 1:**
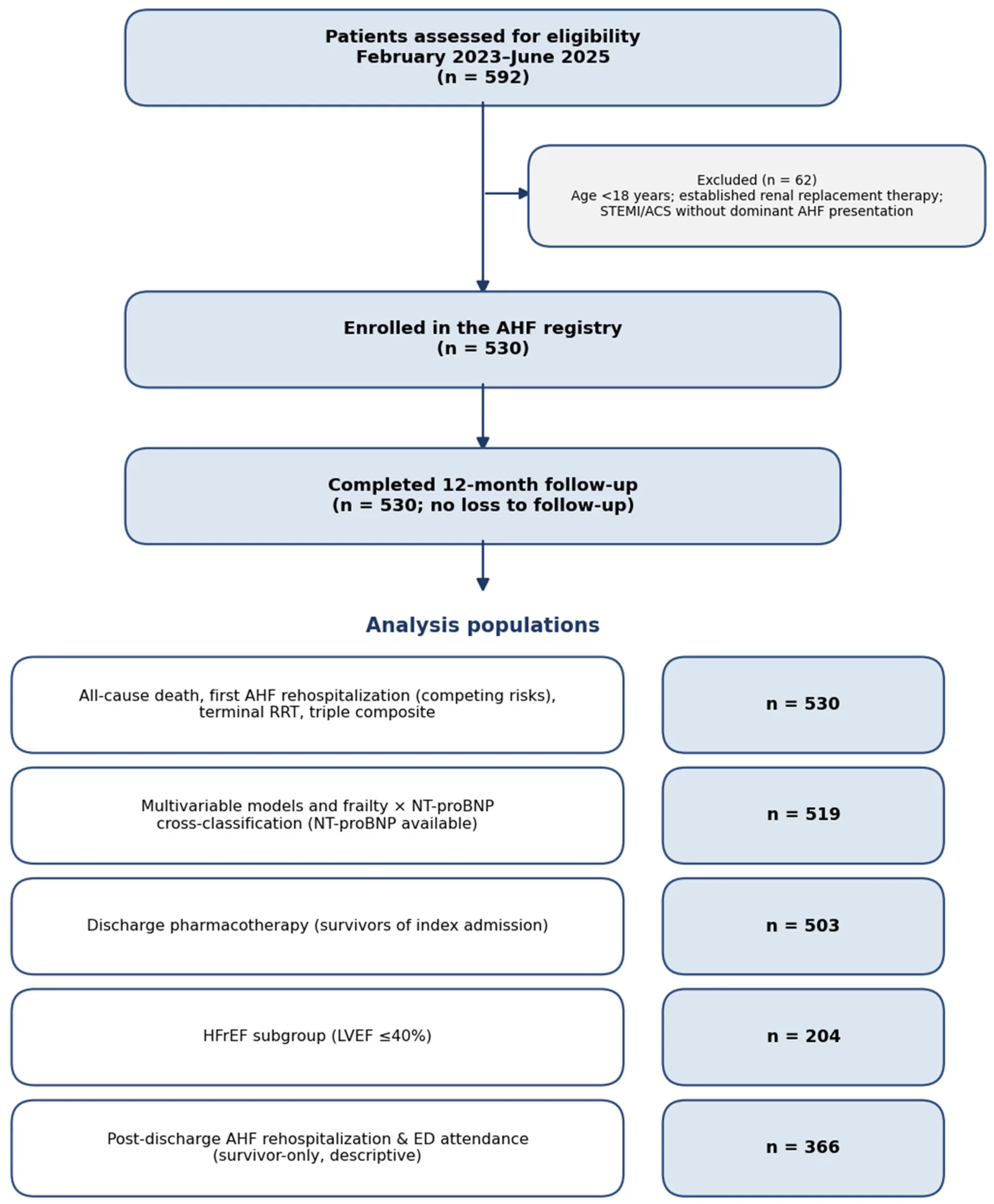
Study flow and analysis populations. AHF, acute heart failure; ACS, acute coronary syndrome; HFrEF, heart failure with reduced ejection fraction; LVEF, left ventricular ejection fraction; RRT, renal replacement therapy; STEMI, ST-elevation myocardial infarction. The competing-risks analysis of first AHF rehospitalization used the full cohort (*n* = 530); *n* = 366 denotes only the survivor subset used for the descriptive post-discharge rehospitalization and emergency-department analyses.

This was a prospective, observational, single-center study conducted at the Department of Cardiology of Venizelio General Hospital, Heraklion, Crete, Greece. Consecutive patients admitted between February 2023 and June 2025 with a primary diagnosis of AHF were enrolled and followed through June 2026. The diagnosis of AHF required the coexistence of clinical signs and symptoms (dyspnea, orthopnea, elevated jugular venous pressure, peripheral edema, pulmonary crackles) together with objective laboratory and imaging findings (elevated natriuretic peptides, evidence of structural cardiac disease, dilated inferior vena cava, B-lines on lung ultrasound), in accordance with the 2021 European Society of Cardiology (ESC) Heart Failure Guidelines [1], with HF classified according to the universal definition [12]. Elevated natriuretic peptides were defined as NT-proBNP ≥ 300 pg/mL, consistent with the acute-setting threshold of the 2021 ESC Guidelines; because natriuretic-peptide concentrations are lower in patients with obesity and may be higher in older patients and in those with atrial fibrillation (AF) or chronic kidney disease (CKD), values were interpreted in the context of body habitus, age, and these comorbidities. Patients younger than 18 years, those already receiving renal replacement therapy (RRT), and those admitted primarily for ST-elevation myocardial infarction or an acute coronary syndrome without a dominant AHF presentation were excluded. Of 592 patients assessed for eligibility, 62 met one or more exclusion criteria and were not enrolled, leaving a final analysis cohort of 530 patients with complete one-year follow-up and no loss to follow-up (Figure 1). Analyses of mortality, terminal RRT and the triple composite used the full cohort (*n* = 530); multivariable models and the frailty–NT-proBNP cross-classification used the 519 patients with an available admission NT-proBNP (the only reference-model predictor with missing data; NT-proBNP was missing in 11 patients [2.1%], while age, admission estimated glomerular filtration rate (eGFR), left ventricular ejection fraction (LVEF) and CFS were complete), analyzed as complete cases; discharge pharmacotherapy was described in the 503 survivors of the index hospitalization, and in the 204 of these with LVEF ≤ 40%; and post-discharge rehospitalization and emergency-department attendance were described in the 366 patients alive and free from terminal RRT at 12 months. All participants provided written informed consent; the study was approved by the Scientific Council of Venizelio General Hospital of Heraklion (5 August 2022; protocol 15/05-08-2022) and conducted in accordance with the Declaration of Helsinki. Data from this registry have previously been used to compare heart failure phenotypes defined by LVEF [13] and to examine multivalvular involvement [14]; frailty was not the primary exposure in either analysis.

### 2.2. Definitions

Right heart failure was defined by the coexistence of at least two of the following: (i) clinical systemic venous congestion (elevated jugular venous pressure, peripheral edema, hepatomegaly or ascites) without a predominant left-sided cause; (ii) echocardiographic right ventricular (RV) dilation and/or systolic dysfunction (tricuspid annular plane systolic excursion [TAPSE] < 17 mm and/or tricuspid annular S’ < 9.5 cm/s); and (iii) elevated right-sided pressures (estimated pulmonary artery systolic pressure [PASP] > 35 mmHg or intermediate or high echocardiographic probability of pulmonary hypertension); isolated right HF denoted right HF without significant left-sided dysfunction. Coronary artery disease was defined as ≥50% stenosis in at least one epicardial vessel or equivalent evidence of ischemia on non-invasive testing. Chronic kidney disease was defined as an eGFR < 60 mL/min/1.73 m^2^ persisting for ≥3 months and staged by the Kidney Disease: Improving Global Outcomes (KDIGO) 2012 classification (Supplementary Table S1). Acute kidney injury was defined, per KDIGO criteria, as an in-hospital increase in serum creatinine of ≥0.3 mg/dL within 48 h or to ≥1.5 times baseline. Worsening renal function (WRF) was graded as an ordinal class from the ratio of peak in-hospital to baseline serum creatinine (class 0, <1.25; 1, 1.25–1.50; 2, 1.51–2.00; 3, 2.01–3.00; 4, >3.00), with in-hospital renal replacement therapy (RRT) constituting the most severe grade (class 5) (Supplementary Table S2). Clinical phenotypes at presentation (acute decompensated HF, acute pulmonary edema, isolated right HF, cardiogenic shock) and significant (moderate or severe) valvular disease were classified according to the 2021 ESC Guidelines and ESC/EACVI recommendations; multivalvular disease denoted at least moderate disease in more than one valve (Supplementary Table S3).

### 2.3. Frailty Assessment

Baseline frailty was graded with the 9-level Clinical Frailty Scale (CFS, version 2.0; Table 1) [8], applied to the patient’s stable functional status in the two weeks preceding admission and thereby capturing pre-morbid rather than acute vulnerability. Patients were classified into three strata—non-frail (CFS 1–3), mildly frail (CFS 4–5) and moderately-to-severely frail (CFS 6–9)—which served as the primary grouping for descriptive and survival comparisons; the binary definition CFS ≥ 5 was used for the frailty–NT-proBNP cross-classification and as a secondary survival contrast, whereas the three-level CFS stratum, entered as an ordinal variable, was used in the primary incremental models. The CFS was assigned at admission by one of two trained cardiologists on the basis of the patient’s pre-morbid functional status, using history obtained directly from the patient together with collateral history from relatives or close caregivers, supplemented where available by prior medical records; assessors were necessarily aware of the admission clinical picture but were blinded to all follow-up outcomes, which were ascertained only later. Only the three-stratum grouping and the CFS ≥ 5 dichotomy were retained in the registry database; individual CFS grades were not stored.

### 2.4. Data Collection

At admission, demographic characteristics (age, sex), primary HF etiology (ischemic/non ischemic), de novo versus acute-on-chronic status, New York Heart Association (NYHA) functional class and a comprehensive comorbidity profile were recorded prospectively. Comorbidities included coronary artery disease, arterial hypertension, AF, CKD, diabetes mellitus, active or prior malignancy, airway disease, stroke/transient ischemic attack, active smoking, and prior device therapy or revascularization. Admission laboratory values included creatinine, estimated glomerular filtration rate (eGFR), albumin, sodium, potassium, urea, uric acid, C-reactive protein, N-terminal pro–B-type natriuretic peptide (NT-proBNP), high-sensitivity troponin I, transaminases, lipid profile, red-cell distribution width (RDW), hemoglobin and white-cell count; peak in-hospital creatinine and nadir eGFR were also recorded. Chronic and discharge HF pharmacotherapy—renin–angiotensin inhibitors/angiotensin receptor–neprilysin inhibitors (ARNI), beta-blockers, mineralocorticoid-receptor antagonists (MRA), sodium–glucose cotransporter-2 inhibitors (SGLT2i) and diuretics—was documented.

### 2.5. Echocardiographic Assessment

Transthoracic echocardiography was performed during the index hospitalization, after initial clinical stabilization (at a mean of 4 days after admission), and interpreted by two separate experienced cardiologists. LVEF was measured by Simpson’s biplane method. Right ventricular systolic function was assessed by TAPSE and tricuspid annular S’ velocity (RV S’), left ventricular diastolic function by the E/e’ ratio, and left atrial volume index was measured and indexed to body surface area. Estimated PASP was derived from the peak tricuspid regurgitation velocity and estimated right atrial pressure. The echocardiographic probability of pulmonary hypertension was graded according to the 2022 ESC/ERS criteria (Supplementary Table S4).

### 2.6. Outcomes

The primary outcome was 12-month all-cause mortality, analyzed in the full cohort. First AHF rehospitalization was a secondary outcome, analyzed in the full cohort within a competing-risks framework in which death and initiation of renal replacement therapy (RRT) were treated as competing events by design, because the post-RRT clinical pathway was considered no longer comparable with the pre-RRT risk state. In particular, once a patient is established on maintenance dialysis, volume status is managed largely within the dialysis unit through regular ultrafiltration, so decompensation is addressed there rather than through discrete HF admissions, and discrete AHF rehospitalizations therefore become difficult to ascertain reliably; in a sensitivity analysis the rehospitalization models were refitted with death as the sole competing event, censoring patients at the initiation of terminal RRT. Follow-up was recorded at fixed checkpoints (3, 6 and 12 months) under a hierarchical rule whereby, when a rehospitalization and a terminal event fell within the same checkpoint interval, only the terminal event was entered in the categorical outcome fields. The cause of every rehospitalization was ascertained from the recorded ICD-10 discharge diagnosis in the electronic health record, so that classification of an admission as AHF-related was based on the coded diagnosis rather than inferred from event timing; the electronic clinical history was available for all rehospitalizations and the full discharge summary for the majority. The recorded rehospitalization field was used where a value was available; where it was blank because the patient had not survived the follow-up interval, the order of events was reconstructed by comparing the time to the first component of the composite endpoint with the time to death or terminal RRT, both recorded in completed weeks, so that a patient whose first composite event preceded death or terminal RRT was classified as having been rehospitalized first. This identified 57 first rehospitalizations in patients who subsequently died (*n* = 55) or initiated terminal RRT (*n* = 2), for whom the 12-month rehospitalization field was therefore blank; 37 of these were documented in an earlier 3- or 6-month checkpoint field and 20 were identified from the recorded event times alone. On this weekly scale, a rehospitalization occurring in the same week as death could not be separated from it and was coded as death, since the endpoint of interest was non-fatal AHF rehospitalization; rehospitalizations preceding death by at least one week were coded as rehospitalization events. This rule is conservative for the rehospitalization endpoint; it is detailed in Supplementary Methods S1, and a sensitivity analysis bounding its effect is reported in Section 3.4. One patient who recovered renal function after transient in-hospital dialysis was recorded as an in-hospital RRT episode but, because RRT was not terminal in this case, remained at risk for rehospitalization and is included in the 366-patient survivor set. Secondary outcomes included terminal RRT, the composite of all-cause death, AHF rehospitalization, or RRT, emergency department (ED) visits, and in-hospital mortality. ED visits were summarized descriptively in the same 366 patients alive and free from terminal RRT at 12 months. In contrast, terminal RRT was assessed in the full cohort and is reported as the observed proportion of patients initiating RRT rather than as a competing-risks cumulative incidence, since subsequent death was not treated as a competing event for this descriptive endpoint. Outcomes were ascertained at 3, 6 and 12 months by combining the electronic health record—which also captures admissions to other institutions with their ICD-10 diagnoses—with direct telephone contact with the patient or a close relative. No patient was lost to follow-up.

### 2.7. Statistical Analysis

Continuous variables are summarized as median [interquartile range] and compared across the three frailty strata by the Kruskal–Wallis test; categorical variables are presented as counts (percentages) and compared by the χ^2^ test, or Fisher’s exact test where expected cell counts were below five. All outcome analyses used a 12-month time horizon. All-cause mortality was estimated with the Kaplan–Meier method and compared across the non-frail, mildly frail and moderately-to-severely frail strata by the log-rank test. Hazard ratios for all-cause mortality were estimated by Cox proportional-hazards regression, with frailty entered as the three-level ordinal stratum in the primary nested models and, as a secondary contrast, as the binary phenotype (CFS ≥ 5). To distinguish all-cause mortality from non-fatal (AHF rehospitalization) events, the 12-month cumulative incidence of first AHF rehospitalization was estimated with the Aalen–Johansen estimator, treating death and RRT as competing events [15], and compared across strata by Gray’s test; cause-specific hazard ratios were obtained from Cox models censoring competing events, and subdistribution hazard ratios from Fine–Gray models. Because follow-up was administratively complete at 52 weeks with no loss to follow-up, inverse-probability-of-censoring weights were uniform, and the subdistribution model was fitted by retaining patients experiencing a competing event in the risk set for the full follow-up period. The proportional-hazards assumption was assessed with scaled Schoenfeld residuals and departures from linearity by adding quadratic terms for age, eGFR and log NT-proBNP (Supplementary Methods S2); the proportional subdistribution hazards assumption of the Fine–Gray model was examined analogously (Supplementary Methods S3). Discordance between frailty and cardiac severity was assessed by cross-classifying patients on frailty (CFS ≥ 5) and natriuretic-peptide burden (NT-proBNP dichotomized at the median, with tertiles used for display) and comparing 12-month all-cause mortality across the concordant and discordant groups. The incremental prognostic value of frailty was quantified with nested Cox models: a cardiorenal base model (age, log NT-proBNP, admission eGFR and LVEF) and the same model with CFS added; the added value was assessed by the change in discrimination (Harrell’s C-index [16], with 95% confidence intervals from 300 bootstrap samples), by optimism-corrected C-indices and a 95% confidence interval for the ΔC-index derived from 500 bootstrap replications, and by the likelihood-ratio test for nested models; for the extended clinical model the corresponding ΔC-index confidence interval was derived from 400 bootstrap replications. Hazard ratios were additionally expressed per 1 standard deviation, the only scale on which predictors measured in different units are directly comparable, and a multiplicative CFS × log NT-proBNP interaction term was tested. The multivariable models were fitted by complete-case analysis (*n* = 519; 149 deaths), owing to missing values in a small number of predictors. Comparisons of baseline characteristics across strata were exploratory and were not adjusted for multiplicity. A two-sided *p* < 0.05 was considered statistically significant. Analyses were performed in Python 3.12 (Python Software Foundation, Wilmington, DE, USA) using lifelines (Kaplan–Meier estimation, the log-rank test, competing-risks cumulative incidence and Cox proportional-hazards regression), SciPy (hypothesis testing) and statsmodels, with pandas and NumPy for data management.

## 3. Results

### 3.1. Frailty Prevalence and the Frail Phenotype

**Figure 2:**
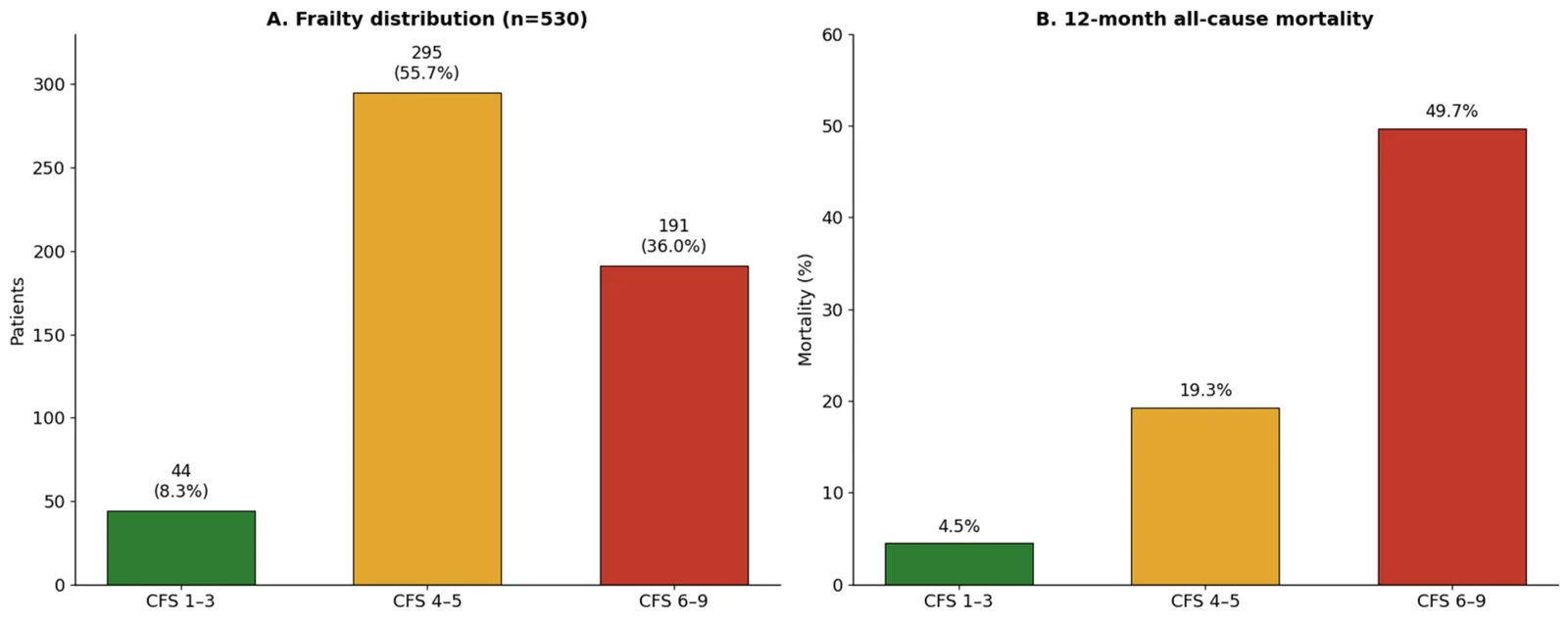
Distribution of frailty (**A**) and 12-month all-cause mortality by frailty stratum (**B**) in the cohort (*n* = 530). CFS, Clinical Frailty Scale.

Frailty was highly prevalent: only 44 patients (8.3%) were non-frail (CFS 1–3), whereas 295 (55.7%) were mildly frail (CFS 4–5) and 191 (36.0%) moderately-to-severely frail (CFS 6–9); two-thirds of the cohort (66.4%) had CFS ≥ 5 (Figure 2A). Increasing frailty delineated a distinct clinical phenotype (Table 2): frailer patients were markedly older (median 66, 78 and 86 years across strata; *p* < 0.001), more often female, and far less likely to present with de novo HF (72.7%, 31.5%, 11.0%; *p* < 0.001). Chronic kidney disease (9.1%, 47.5%, 68.1%), right HF (34.1%, 35.9%, 55.0%) and multivalvular disease (11.4%, 27.8%, 51.3%) all rose steeply with frailty (all *p* < 0.001), as did baseline NYHA class (*p* < 0.001).

### 3.2. Laboratory and Echocardiographic Profile

The frail phenotype was mirrored in the laboratory and echocardiographic profile (Table 3). Frailer patients had higher NT-proBNP (median 3314, 4934 and 6498 pg/mL; *p* < 0.001), urea and uric acid, and lower hemoglobin (13.4, 12.0 and 10.9 g/dL; *p* < 0.001), albumin, eGFR and total cholesterol (all *p* < 0.001), consistent with a congested, anemic, nutritionally depleted and renally impaired state. Echocardiographically, frailty was associated with higher estimated PASP and E/e′, larger left atrial volume, lower TAPSE and RV S′, and a markedly higher probability of pulmonary hypertension (high-probability 22.7%, 46.8% and 69.6%; *p* < 0.001), whereas LVEF rose modestly across strata (median 35%, 45% and 50%; *p* = 0.007), indicating that frailty was not associated with lower LVEF.

### 3.3. In-Hospital Course, Pharmacotherapy and Follow-Up Outcomes

Frailty was associated with a more complicated hospitalization (Table 4): the need for inotropes/vasopressors (2.3%, 6.8%, 20.9%), acute kidney injury (20.5%, 45.4%, 58.6%), in-hospital RRT and in-hospital mortality (0%, 2.7%, 9.9%) all increased with frailty (all *p* ≤ 0.001), as did length of stay. Notably, guideline-directed medical therapy at discharge showed the opposite gradient, decreasing with frailty across all four disease-modifying drug classes—renin–angiotensin-system inhibition with ARNI or ACEi/ARB, of which ARNI specifically fell (40.9%, 10.8%, 3.5%), beta-blockers (86.4%, 84.3%, 64.5%), MRA (79.5%, 58.9%, 51.7%) and SGLT2i (90.9%, 68.6%, 50.0%; all *p* < 0.001 except MRA, *p* = 0.004)—so that the frailest patients, at highest risk, received the least disease-modifying therapy. The same gradient was evident when the analysis was restricted to the 204 survivors with LVEF ≤ 40%, in whom all four classes are formally indicated: discharge prescription of ARNI, MRA and SGLT2i all declined across frailty strata (all *p* ≤ 0.002; Table 4).

### 3.4. Mortality and Heart-Failure Rehospitalization Across Frailty Strata

**Figure 3:**
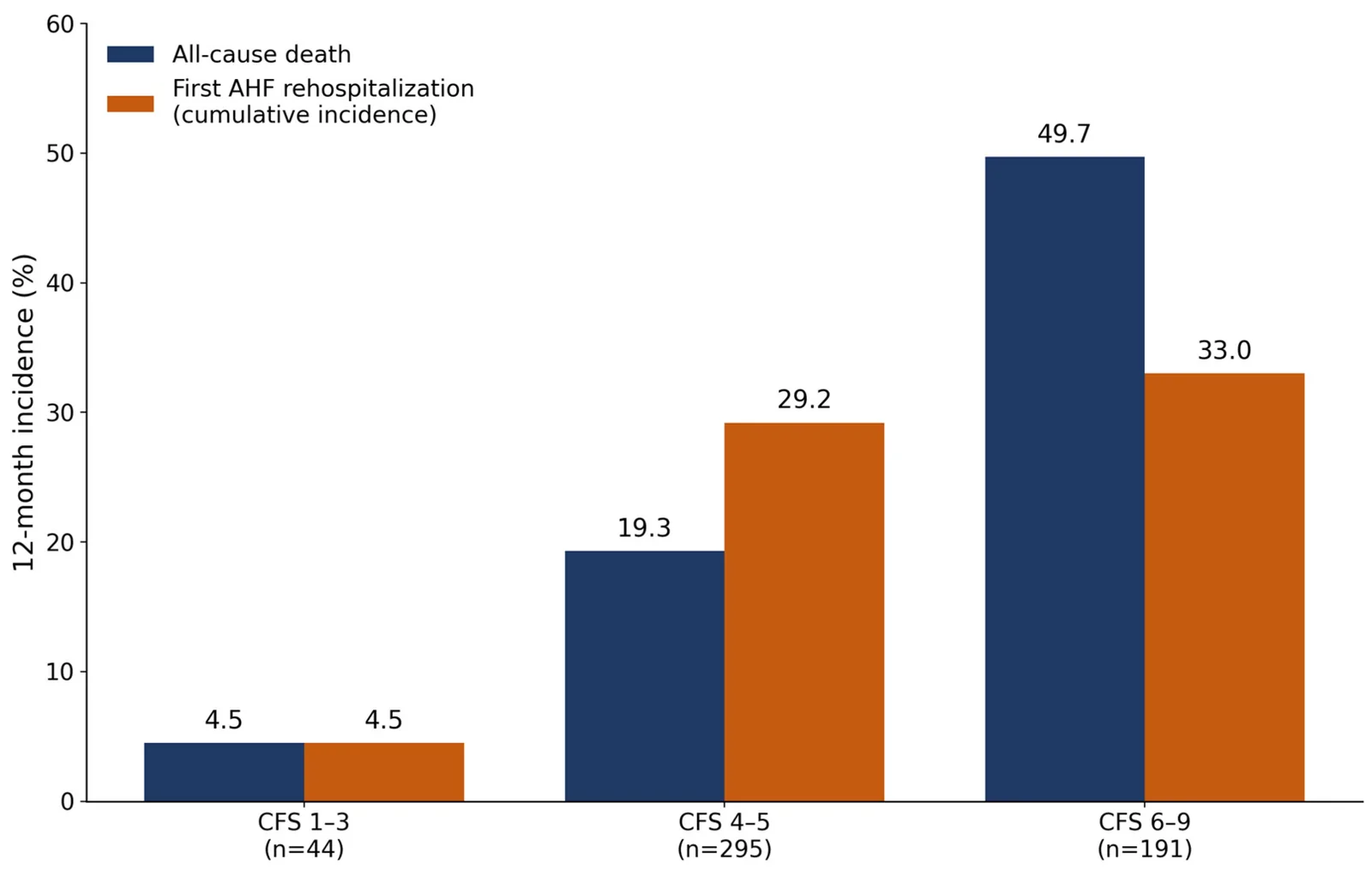
Twelve-month all-cause mortality and cumulative incidence of first AHF rehospitalization (Aalen–Johansen estimator, with death and RRT as competing events), both shown as 12-month estimates in the full cohort by frailty stratum. Both increase with frailty, mortality far more steeply. AHF, acute heart failure; CFS, Clinical Frailty Scale.

Mortality rose steeply and almost monotonically across frailty strata (4.5%, 19.3%, 49.7%; log-rank *p* < 0.001; hazard ratio 3.29 per stratum, 95% CI 2.43–4.45, and 3.81 for CFS ≥5 versus <5) (Table 5, Supplementary Figure S1). The 12-month cumulative incidence of first AHF rehospitalization, with death and RRT as competing events, also increased with frailty (4.5%, 29.2%, 33.0%; Gray’s test *p* < 0.001) (Figure 3), but the absolute increase across strata was smaller than for mortality. The cause-specific hazard ratio for rehospitalization was 1.86 per stratum (95% CI 1.42–2.45), and the subdistribution hazard ratio 1.51 (95% CI 1.19–1.92); these estimates are reported separately from the mortality hazard ratio and should not be directly compared, because they arise from different risk sets and estimands. With death as the only competing event, the cumulative incidence of first AHF rehospitalization was essentially unchanged (4.5%, 29.3% and 33.7% across strata; Gray’s test *p* < 0.001), the cause-specific hazard ratio was essentially unchanged (1.84, 95% CI 1.40–2.42), and the subdistribution hazard ratio marginally higher (1.57, 95% CI 1.23–2.00), confirming robustness to the treatment of RRT (Supplementary Methods S4). In a bounding sensitivity analysis in which all 67 post-discharge deaths without a separately timed antecedent event were reclassified as terminal rehospitalizations—an extreme upper limit for under-ascertainment—the cause-specific hazard ratio for rehospitalization rose only to 2.11 (95% CI 1.67–2.66) (Supplementary Table S5; an alternative, more conservative classification is shown in Supplementary Table S6). Terminal RRT (0%, 1.7%, 9.4%) and the triple composite (9.1%, 42.0%, 68.1%) likewise rose with frailty, whereas emergency-department visits did not differ significantly across strata (47.6%, 65.8%, 66.7%; *p* = 0.064) (Table 5).

### 3.5. Prognostic Value of Frailty Beyond Natriuretic-Peptide Elevation

**Figure 4:**
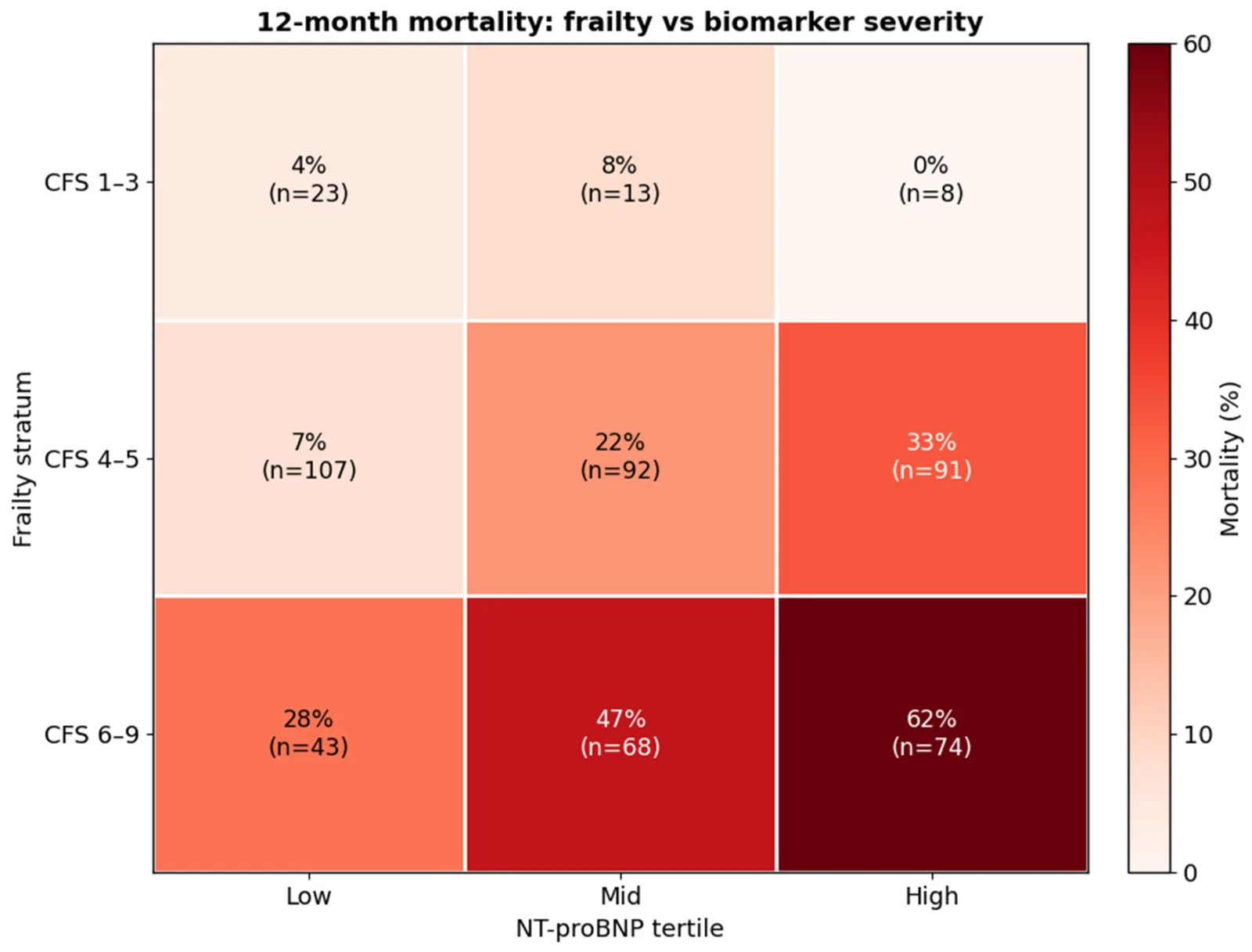
Twelve-month mortality across the joint distribution of frailty stratum and NT-proBNP tertile. Frailty adds prognostic information complementary to biomarker severity; the CFS × log NT-proBNP interaction was not significant (*p* = 0.91). *n* = 519 with available NT-proBNP. CFS, Clinical Frailty Scale.

Frailty carried prognostic information complementary to natriuretic-peptide elevation (Table 6, Figure 4). When patients were cross-classified by frailty and NT-proBNP (median 5248 pg/mL), frail patients with below-median NT-proBNP had 12-month mortality comparable to that of patients with CFS < 5 and above-median NT-proBNP (35/144, 24.3%, 95% CI 18.0–31.9 vs. 13/59, 22.0%, 95% CI 13.4–34.1; overlapping confidence intervals). Mortality was lowest in CFS <5/low-NT-proBNP patients (8/116, 6.9%; 95% CI 3.5–13.0) and highest in frail/high-NT-proBNP patients (93/200, 46.5%; 95% CI 39.7–53.4). CFS and log NT-proBNP were only weakly correlated (Spearman ρ = 0.20, *p* < 0.001) and no multiplicative interaction between CFS and log NT-proBNP was detected (*p* = 0.91) (Figure 4).

### 3.6. Incremental Prognostic Value of Frailty

**Figure 5:**
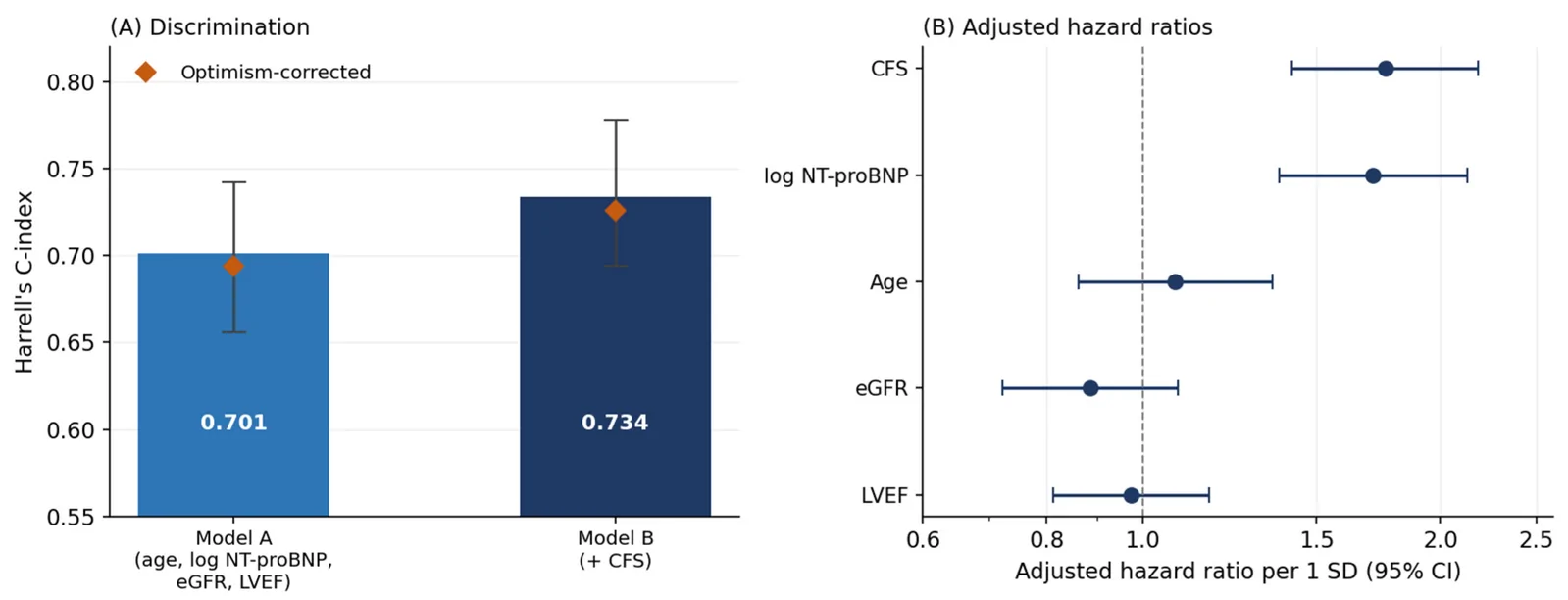
Incremental value of frailty. (**A**) Harrell C-index for the cardiorenal model with and without CFS (bars, apparent estimate with bootstrap 95% CI; diamonds, optimism-corrected estimate). (**B**) Adjusted hazard ratios per 1 standard deviation from the multivariable model for 1-year mortality, plotted on a logarithmic scale. CFS, Clinical Frailty Scale.

Frailty added discrimination beyond a cardiorenal reference model (Table 7, Figure 5). Adding CFS to a model containing age, log NT-proBNP, admission eGFR and LVEF increased the Harrell C-index from 0.701 (95% CI 0.656–0.742) to 0.734 (95% CI 0.694–0.778) and significantly improved model fit (likelihood-ratio χ^2^ = 27.7, *p* < 0.001). After bootstrap optimism correction the C-indices were 0.694 and 0.726, with an optimism-corrected ΔC-index of 0.032 (apparent ΔC-index 0.033, paired bootstrap 95% CI 0.007–0.063). Apparent calibration in the development cohort was satisfactory (apparent calibration slope 1.00; Supplementary Figure S2 shows the apparent calibration plot). The 12-month Brier score was 0.166 versus 0.205 for the null model (scaled Brier 0.19); no censoring adjustment was required, as 12-month status was known for every patient. These estimates represent apparent, internal performance in the development cohort; optimism-corrected calibration was not formally derived, and external validation is required before any clinical use. Frailty remained strongly and independently associated with mortality after adjustment (adjusted hazard ratio 2.54 per stratum, 95% CI 1.78–3.62). Expressed per 1 standard deviation, the hazard ratio for CFS was 1.76 (95% CI 1.42–2.18) and that for log NT-proBNP 1.71 (95% CI 1.38–2.13) (Figure 5B). Entering CFS as a categorical variable—the pre-specified three strata—gave hazard ratios of 3.32 (95% CI 0.78–14.20) for CFS 4–5 and 8.25 (95% CI 1.85–36.74) for CFS 6–9 relative to CFS 1–3. These categorical estimates, which do not assume an equal increment between adjacent strata, are wide because only two deaths occurred in the CFS 1–3 reference group (*n* = 44); the three-category hazard ratios should be regarded as descriptive, and the dichotomous CFS ≥ 5 parameterization, being statistically more stable, provides the more reliable estimate of the frailty–mortality association (binary CFS ≥ 5 contrast: hazard ratio 3.81, 95% CI 2.40–6.04); the ordinal hazard ratio should be read as a summary of the across-strata trend. In a fuller clinical model additionally adjusted for sex, de novo versus chronic HF, baseline NYHA class, admission sodium and albumin (513 complete cases with 147 deaths, owing to additional missing values in albumin), frailty remained independently associated with mortality (adjusted hazard ratio 1.90 per stratum, 95% CI 1.25–2.90; *p* = 0.003) and continued to add discrimination (ΔC-index 0.010, 95% CI 0.001–0.027; Supplementary Table S7). The proportional-hazards assumption held for every covariate (all *p* ≥ 0.18) and no departure from linearity was detected for age, eGFR or log NT-proBNP (all *p* ≥ 0.30); full diagnostics are given in Supplementary Methods S2.

## 4. Discussion

### 4.1. Principal Findings

In a prospective cohort of 530 patients hospitalized for AHF, frailty was highly prevalent and was a strong, multisystem prognostic marker, consistent with the high burden and adverse prognosis of frailty reported across heart-failure populations [3,6,7]. Four findings stand out. First, increasing frailty delineated a coherent phenotype of older, congested, anemic, renally impaired patients with prominent right-heart and valvular involvement and, notably, higher rather than lower LVEF. Second, frailty was associated with both mortality and HF rehospitalization, but the absolute increase across strata was greater for mortality than for documented non-fatal AHF rehospitalization; because the hazard ratios for the two endpoints arise from different risk sets and estimands, we base this contrast on the cumulative-incidence pattern rather than on a direct comparison of hazard ratios. Third, frailty carried prognostic information complementary to natriuretic-peptide elevation, with frail patients showing higher mortality than their NT-proBNP level alone would predict [10]. Fourth, frailty added discrimination beyond a cardiorenal reference model, although the increment beyond a fuller clinical model was modest.

### 4.2. The Frail Phenotype

The characteristics that intensified with frailty—advanced age, female preponderance, chronic kidney disease, anemia, hypoalbuminemia, right-heart failure, multivalvular disease and pulmonary hypertension—describe a multisystem, low-reserve phenotype rather than a more severe form of left ventricular dysfunction, in line with the universal definition’s emphasis on HF as a systemic syndrome [12] and with the multimorbid profile documented in contemporary AHF registries [17,18]. The direction of the LVEF gradient reinforces this: median LVEF was higher, not lower, with increasing frailty, so that frailty in AHF tracks a preserved-ejection-fraction, multimorbid phenotype rather than more severe systolic dysfunction, aligning with reports that frailty is at least as prevalent, and as prognostically important, in HF with preserved as with reduced ejection fraction [19].

### 4.3. Stronger Association with Mortality than with Documented Non-Fatal AHF Rehospitalization

Frailty was associated with both all-cause mortality and documented non-fatal AHF rehospitalization. Mortality rose more than tenfold across frailty strata, whereas the cumulative incidence of first AHF rehospitalization rose approximately sevenfold and then plateaued between the two frailer strata. The absolute cumulative incidence increased more steeply across frailty strata for mortality than for documented non-fatal AHF rehospitalization, an asymmetry that is easily obscured when death is not treated as a competing event [15]. The divergence is compatible with death progressively removing the frailest patients from the population at risk of a non-fatal event. The clinical corollary is that frailty is not a purely cardiac risk marker: it was associated with higher mortality and greater global vulnerability alongside HF events—in keeping with the strong, graded relationship between frailty and mortality described in earlier cohorts [6,7]—supporting consideration of holistic assessment and comprehensive geriatric input in addition to HF-directed therapy.

### 4.4. Complementarity with Biomarker Severity

Frailty carried prognostic information that was complementary to, and only weakly correlated with, natriuretic-peptide elevation (ρ = 0.20). Notably, patients combining high frailty and high NT-proBNP had the highest mortality, indicating that the two markers are additive rather than contradictory. The difference between these two cells is modest and should be read as illustrating complementarity rather than as evidence that frailty outweighs natriuretic-peptide elevation. Risk stratification anchored solely on natriuretic peptides or hemodynamic congestion may therefore overlook clinically important risk associated with frailty in patients whose natriuretic peptides are only modestly elevated.

A pathophysiological reading helps explain this complementarity. Natriuretic peptides index a single dimension—myocardial wall stress and hemodynamic congestion—whereas frailty reflects depletion of physiological reserve across multiple systems, so the two can diverge. In HF the underlying biology is thought to overlap and compound, although these pathways were not measured in the present study: chronic systemic inflammation, sustained neurohormonal activation with anabolic–catabolic uncoupling, sarcopenia, endocrine dysregulation and chronic cerebral hypoperfusion are all hypothesized to erode the capacity to withstand and recover from an acute decompensation [5,11]. Consistent with this multisystem view, renal–endothelial stress indices such as the Endothelial Activation and Stress Index (EASIX) and inflammation-based scores such as the Scottish inflammatory prognostic score have been independently associated with adverse in-hospital outcomes in acute HF [20,21], situating frailty within the broader interplay of renal dysfunction, endothelial stress, inflammation and functional vulnerability. A frail patient with only modestly elevated NT-proBNP may therefore already have limited physiological reserve, part of which is not captured by natriuretic peptides—consistent with our finding that frailty was more strongly associated with mortality than with documented non-fatal HF events. Because cause of death was not adjudicated, we cannot determine what proportion of this mortality was cardiovascular, and the association should not be read as evidence of a specifically non-cardiac mechanism. Frailty and natriuretic peptides thus mark different, only partly overlapping axes of risk, and are best read together rather than one in place of the other, reinforcing recommendations that frailty be assessed alongside conventional risk markers in HF [4].

### 4.5. Incremental Value and Clinical Implications

Frailty improved discrimination beyond age, natriuretic peptides, renal function and ejection fraction, with an adjusted hazard per standard deviation comparable to that of log NT-proBNP (1.76 versus 1.71)—a similar adjusted association for a bedside, judgement-based score requiring no laboratory input. The incremental discrimination attributable to CFS was smaller when tested against a fuller clinical model (ΔC-index 0.010) than against the four-variable cardiorenal model (ΔC-index 0.033); the gain beyond a comprehensive clinical assessment was therefore statistically significant but modest (Supplementary Table S7). Coupled with the observation that the frailest patients received the least guideline-directed therapy at discharge [22,23], these data argue for routine, structured frailty assessment at admission [24], both to refine prognosis and to prompt individualized—rather than nihilistic—therapeutic decisions in this vulnerable group [25,26,27].

This inverse relationship between frailty and disease-modifying therapy deserves emphasis. The frailest patients—those at highest mortality risk—were the least likely to be discharged on renin–angiotensin inhibition, a beta-blocker, a mineralocorticoid-receptor antagonist or an SGLT2i, a pattern that has been described as a risk–treatment paradox in frail HF populations, in whom guideline-directed drugs are frequently under-prescribed and seldom up-titrated [23]. This lower prescription rate may reflect contraindications, intolerance, clinical complexity, treatment preferences or therapeutic inertia, and the present observational analysis cannot distinguish among these explanations. Reasons for non-prescription—such as eligibility, intolerance, hypotension, renal dysfunction, hyperkalemia and goals of care—were not comprehensively recorded, so this gradient is reported as a descriptive association and not as evidence of undertreatment or of a causal risk–treatment paradox. External evidence nonetheless suggests that frailty alone is not a sufficient reason to withhold therapy: a systematic review of heart-failure treatment stratified by frailty reported that the efficacy and safety of guideline-directed therapy are broadly maintained across frailty strata [22], and SGLT2i confer consistent benefit across a wide spectrum of patients with HF [25]. Taken together, this external evidence argues against treating frailty as a reason to withhold therapy and supports structured frailty assessment coupled with a deliberate attempt at guideline-directed therapy rather than its withdrawal, although our observational study did not itself evaluate treatment effect [22,23,25]. Importantly, the prescribing gradient persisted when the analysis was restricted to treatment-eligible patients with LVEF ≤ 40%, among whom discharge prescription of ARNI, MRA and SGLT2i all declined across frailty strata (all *p* ≤ 0.002; Table 4), so this gradient cannot be attributed to the greater proportion of preserved ejection fraction among frail patients.

### 4.6. Limitations

This is a single-center study of an elderly Mediterranean cohort, which may limit generalizability. The cohort was old (median age 81 years) and predominantly frail (only 8.3% non-frail); the observed frailty distribution and effect estimates should therefore not be assumed to represent the broader AHF population, and external validation in multicenter cohorts with different age structures, HF phenotypes and health systems is required. Frailty was assessed once, at baseline, and dynamic changes were not captured. Cause of death was not formally adjudicated, so the interpretation of mortality rests on the divergence from HF events rather than on adjudicated non-cardiovascular death. The lower use of guideline-directed therapy in frail patients is observational and subject to confounding by indication. Event timing was available in completed weeks rather than as exact dates, so a rehospitalization occurring in the same week as death could not be distinguished from it and was coded as death; the reported cumulative incidence of rehospitalization is therefore a conservative estimate, although in the bounding analysis (Section 3.4) the steeper mortality gradient was reversed only under the deliberately extreme assumption that every ambiguous death was preceded by a terminal readmission. Only the three-level CFS grouping and the CFS ≥ 5 dichotomy were retained in the registry database, so analyses by individual CFS grade could not be performed, and patients graded CFS 9 could not be identified or excluded; since CFS 9 incorporates an expected survival of less than six months, some incorporation bias in its association with 12-month mortality cannot be excluded, and for this reason we describe CFS as associated with, rather than as predicting, mortality. Such patients are, however, likely to have been few, since in routine practice individuals with an overtly terminal trajectory are more often admitted to internal-medicine or palliative-care services than to a cardiology ward. CFS was assigned prospectively at admission by one of two cardiologists, and although grading followed a standard description, formal inter-rater reliability was not assessed. Standardized delirium or cognitive screening was not systematically performed, and the frailty score relied on pre-admission functional history rather than a formal cognitive assessment; anchoring the score to the patient’s habitual baseline through collateral history mitigates, but does not fully exclude, misclassification arising from acute cognitive change. Comparisons across the many baseline characteristics were exploratory and not corrected for multiplicity. The non-frail reference stratum was small (*n* = 44) with few deaths, so categorical mortality estimates relative to it are imprecise, with correspondingly wide confidence intervals. Finally, as in any observational study, residual confounding cannot be excluded.

## 5. Conclusions

In elderly patients hospitalized for AHF, frailty is highly prevalent and is a strong, multisystem prognostic marker: it defines a low-reserve, multimorbid phenotype and was more strongly associated with all-cause mortality than with documented non-fatal HF rehospitalization, carried prognostic information complementary to natriuretic-peptide elevation, and improved risk discrimination beyond four cardiorenal variables. These findings support consideration of structured frailty assessment to complement biomarker- and imaging-based stratification; whether frailty-guided management improves outcomes requires prospective evaluation.

## Figures and Tables

**Table 1:** The Clinical Frailty Scale (CFS) used to grade baseline frailty. CFS, Clinical Frailty Scale.

CFS	Category	Description
1	Very fit	Robust, active, energetic and motivated; typically exercise regularly and are among the fittest for their age.
2	Fit	No active disease symptoms but less fit than category 1; active occasionally, e.g., seasonally.
3	Managing well	Medical problems are well controlled, even if occasionally symptomatic, but not regularly active beyond routine walking.
4	Living with very mild frailty	Not dependent on others for daily help, but symptoms often limit activities; commonly “slowed up” and/or tired during the day.
5	Living with mild frailty	More evident slowing; need help with higher-order instrumental activities (finances, transport, heavy housework, medications).
6	Living with moderate frailty	Need help with all outside activities and with keeping house; often problems with stairs, and need help with bathing and possibly dressing.
7	Living with severe frailty	Completely dependent for personal care, from any cause (physical or cognitive), yet stable and not at high risk of dying.
8	Living with very severe frailty	Completely dependent for personal care and approaching end of life; typically could not recover even from a minor illness.
9	Terminally ill	Approaching the end of life, with a life expectancy of under six months, who are not otherwise living with severe frailty.

**Table 2:** Demographic characteristics and comorbidities across frailty strata. Values are *n* (%) unless otherwise stated; continuous variables are median [IQR]. *p*-values are for comparison across the three strata (Kruskal–Wallis, χ^2^ or Fisher’s exact test, as appropriate). CABG, coronary artery bypass grafting; CFS, Clinical Frailty Scale; HF, heart failure; ICD, implantable cardioverter-defibrillator; NYHA, New York Heart Association; TIA, transient ischemic attack.

Parameter	Total (*n* = 530)	CFS 1–3 (*n* = 44)	CFS 4–5 (*n* = 295)	CFS 6–9 (*n* = 191)	*p*
**Demographics**					
Age (years)	81 [74–86]	66 [58–73]	78 [73–84]	86 [82–89]	<0.001
Male sex	284 (53.6)	24 (54.5)	181 (61.4)	79 (41.4)	<0.001
De novo heart failure	146 (27.5)	32 (72.7)	93 (31.5)	21 (11.0)	<0.001
**Comorbidities**					
Ischemic etiology	167 (31.5)	11 (25.0)	110 (37.3)	46 (24.1)	0.001
Atrial fibrillation	313 (59.1)	21 (47.7)	168 (56.9)	124 (64.9)	0.061
Diabetes mellitus	275 (51.9)	17 (38.6)	155 (52.5)	103 (53.9)	0.177
Arterial hypertension	462 (87.2)	37 (84.1)	265 (89.8)	160 (83.8)	0.122
Airway disease	169 (31.9)	16 (36.4)	95 (32.2)	58 (30.4)	0.732
Chronic kidney disease	274 (51.7)	4 (9.1)	140 (47.5)	130 (68.1)	<0.001
Prior stroke/TIA	39 (7.4)	0 (0.0)	21 (7.1)	18 (9.4)	0.095
Active malignancy	51 (9.6)	3 (6.8)	26 (8.8)	22 (11.5)	0.494
Previous malignancy	36 (6.8)	2 (4.5)	24 (8.1)	10 (5.2)	0.382
Coronary artery disease No Yes Undefined	116 (21.9) 238 (44.9) 176 (33.2)	19 (43.2) 17 (38.6) 8 (18.2)	62 (21.0) 149 (50.5) 84 (28.5)	35 (18.3) 72 (37.7) 84 (44)	<0.001
Prior CABG	62 (11.7)	0 (0.0)	40 (13.6)	22 (11.5)	0.033
Prior valve replacement	33 (6.2)	2 (4.5)	19 (6.4)	12 (6.3)	0.888
ICD	31 (5.8)	0 (0.0)	25 (8.5)	6 (3.1)	0.011
Permanent pacemaker	51 (9.6)	2 (4.5)	26 (8.8)	23 (12.0)	0.245
Right heart failure	226 (42.6)	15 (34.1)	106 (35.9)	105 (55.0)	<0.001
**Baseline functional status**					
Baseline NYHA class I II III IV	22 (4.2) 103 (19.4) 321 (60.6) 84 (15.8)	22 (50.0) 22 (50.0) 0 (0.0) 0 (0.0)	0 (0.0) 81 (27.5) 213 (72.2) 1 (0.3)	0 (0.0) 0 (0.0) 108 (56.5) 83 (43.5)	<0.001
**Clinical phenotype at presentation**					
Acute decompensated HF	389 (73.4)	35 (79.5)	211 (71.5)	143 (74.9)	0.451
Acute pulmonary edema	133 (25.1)	9 (20.5)	81 (27.5)	43 (22.5)	0.357
Cardiogenic shock	6 (1.1)	0 (0.0)	1 (0.3)	5 (2.6)	0.052
Isolated right HF	2 (0.4)	0 (0.0)	2 (0.7)	0 (0.0)	0.449
**Valvular disease (moderate/severe)**					
Mitral regurgitation	227 (42.8)	14 (31.8)	113 (38.3)	100 (52.4)	0.003
Tricuspid regurgitation	232 (43.8)	9 (20.5)	106 (35.9)	117 (61.3)	<0.001
Aortic stenosis	105 (19.8)	2 (4.5)	50 (16.9)	53 (27.7)	<0.001
Aortic regurgitation	67 (12.6)	2 (4.5)	28 (9.5)	37 (19.4)	0.001
Mitral stenosis	22 (4.2)	1 (2.3)	9 (3.1)	12 (6.3)	0.176
Multivalvular disease	185 (34.9)	5 (11.4)	82 (27.8)	98 (51.3)	<0.001

**Table 3:** Admission laboratory and echocardiographic parameters across frailty strata. Continuous variables are median [IQR]; *p*-values by Kruskal–Wallis (continuous), χ^2^ or Fisher’s exact test (categorical). CRP, C-reactive protein; eGFR, estimated glomerular filtration rate; E/e′, ratio of early mitral inflow to mitral annular velocity; LAVi, left atrial volume index; LVEF, left ventricular ejection fraction; PASP, pulmonary artery systolic pressure; RDW, red-cell distribution width; RV, right ventricle; TAPSE, tricuspid annular plane systolic excursion; TSH, thyroid-stimulating hormone; WBC, white blood cell count.

Parameter	Total (*n* = 530)	CFS 1–3 (*n* = 44)	CFS 4–5 (*n* = 295)	CFS 6–9 (*n* = 191)	*p*
**Admission laboratory & vitals**					
Systolic BP (mmHg)	145 [127–170]	145 [130–170]	150 [130–170]	140 [120–170]	0.078
Baseline eGFR (mL/min/1.73 m^2^)	58 [43–77]	83 [75–94]	63 [46–77]	49 [36–64]	<0.001
Admission eGFR (mL/min/1.73 m^2^)	57 [40–75]	79 [71–92]	60 [44–78]	47 [32–62]	<0.001
Admission creatinine (mg/dL)	1.14 [0.92–1.53]	0.92 [0.79–1.06]	1.13 [0.89–1.49]	1.23 [1.02–1.73]	<0.001
Admission urea (mg/dL)	56 [43–82]	40 [34–47]	52 [42–72]	70 [53–105]	<0.001
NT-proBNP (pg/mL)	5248 [3032–10,533]	3314 [1576–6874]	4934 [2902–9721]	6498 [3857–13,634]	<0.001
Troponin (pg/mL)	29 [14–69]	27 [12–69]	28 [14–67]	31 [15–79]	0.336
CRP (mg/L)	0.9 [0.4–2.2]	0.8 [0.4–2.3]	0.9 [0.4–2.0]	1.0 [0.4–2.5]	0.637
WBC (×10^3^/µL)	8.6 [6.8–11.1]	9.2 [7.0–11.3]	8.8 [7.0–11.1]	8.3 [6.5–10.7]	0.181
Neutrophil-to-lymphocyte ratio	4.2 [2.9–6.4]	3.3 [2.5–4.8]	4.3 [2.8–6.2]	4.2 [3.1–7.5]	0.018
Albumin (g/dL)	4.0 [3.8–4.3]	4.2 [3.9–4.3]	4.1 [3.9–4.3]	3.9 [3.7–4.1]	<0.001
Hemoglobin (g/dL)	11.7 [10.4–13.0]	13.4 [12.2–14.5]	12.0 [10.6–13.0]	10.9 [9.8–12.4]	<0.001
RDW (%)	16.2 [15.0–18.3]	14.7 [14.1–16.3]	16.1 [14.8–17.9]	16.5 [15.4–18.9]	<0.001
Sodium (mEq/L)	139 [137–141]	139 [137–141]	139 [137–141]	138 [136–141]	0.058
Potassium (mEq/L)	4.5 [4.1–4.9]	4.4 [4.2–4.8]	4.5 [4.1–4.8]	4.6 [4.2–5.0]	0.016
Uric acid (mg/dL)	7.5 [6.1–9.1]	6.6 [5.2–7.7]	7.4 [6.1–8.8]	8.1 [6.7–10.1]	<0.001
HbA1c (%)	6.1 [5.6–6.9]	6.0 [5.5–6.8]	6.1 [5.6–6.8]	6.1 [5.6–7.3]	0.385
Glucose (mg/dL)	132 [109–180]	122 [107–180]	133 [109–182]	134 [110–178]	0.521
Total cholesterol (mg/dL)	134 [110–161]	152 [136–185]	135 [110–163]	126 [105–152]	<0.001
LDL (mg/dL)	73 [55–96]	94 [81–126]	73 [55–98]	68 [52–86]	<0.001
HDL (mg/dL)	37 [30–47]	36 [33–45]	37 [30–47]	37 [30–45]	0.761
Triglycerides (mg/dL)	101 [77–130]	115 [83–142]	104 [81–130]	90 [74–127]	0.024
Ferritin (ng/mL)	55 [30–118]	60 [28–120]	56 [33–122]	51 [27–102]	0.293
TSH (µIU/mL)	1.22 [0.72–2.09]	1.16 [0.73–1.93]	1.23 [0.69–2.05]	1.21 [0.77–2.30]	0.714
**Echocardiographic parameters**					
LVEF (%)	45 [30–55]	35 [25–51]	45 [30–55]	50 [35–55]	0.007
Estimated PASP (mmHg)	40 [35–55]	35 [30–45]	40 [30–50]	50 [40–60]	<0.001
E/e′	16.5 [13.0–23.0]	14.0 [11.0–18.8]	16.0 [13.0–21.0]	19.0 [15.0–25.0]	<0.001
RV S′ (cm/s)	10.0 [9.0–12.0]	11.0 [9.8–13.0]	11.0 [9.0–12.2]	10.0 [8.0–11.0]	<0.001
TAPSE (mm)	18 [15–19]	18 [16–20]	18 [16–20]	17 [15–19]	0.001
LAVi (ml/m^2^)	51 [44–63]	46 [40–55]	50 [43–62]	54 [46–70]	<0.001
Pulmonary hypertension probability					<0.001
Low Intermediate High	75 (14.2) 174 (32.8) 281 (53.0)	13 (29.5) 21 (47.7) 10 (22.7)	52 (17.6) 105 (35.6) 138 (46.8)	10 (5.2) 48 (25.1) 133 (69.6)	
LV dilation	135 (25.5)	16 (36.4)	87 (29.5)	32 (16.8)	0.002
RV dilation	130 (24.5)	8 (18.2)	59 (20.0)	63 (33.0)	0.003

**Table 4:** In-hospital course, pharmacotherapy and follow-up outcomes across frailty strata. Values are *n* (%) unless stated. Discharge medications are reported among the 503 survivors of the index hospitalization. Three- and six-month mortality and the six-month combined endpoint are cumulative outcomes in the full cohort (*n* = 530). Six-month ED visits and AHF readmissions are reported among the 422 patients evaluable for these outcomes; six-month terminal RRT, like the mortality endpoints, is a cumulative outcome in the full cohort (*n* = 530); and the 12-month rehospitalization and emergency-department analyses used the 366 patients alive and free from terminal RRT at 12 months. The HFrEF subgroup comprises the 204 survivors with LVEF ≤ 40%, in whom all four disease-modifying drug classes are formally indicated. ARNI, angiotensin receptor–neprilysin inhibitor; ED, emergency department; MRA, mineralocorticoid receptor antagonist; RRT, renal replacement therapy; SGLT2i, sodium–glucose cotransporter-2 inhibitor; WRF, worsening renal function.

Parameter	Total (*n* = 530)	CFS 1–3 (*n* = 44)	CFS 4–5 (*n* = 295)	CFS 6–9 (*n* = 191)	*p*
**In-hospital course**					
Inotropes/vasopressors	61 (11.5)	1 (2.3)	20 (6.8)	40 (20.9)	<0.001
Acute kidney injury	255 (48.1)	9 (20.5)	134 (45.4)	112 (58.6)	<0.001
RRT during hospitalization	13 (2.5)	0 (0.0)	2 (0.7)	11 (5.8)	0.001
Worsening renal function class					0.001
WRF 0 WRF 1 WRF 2 WRF 3 WRF 4 WRF 5 (in-hospital RRT)	267 (50.4) 133 (25.1) 90 (17.0) 25 (4.7) 2 (0.4) 13 (2.5)	31 (70.5) 7 (15.9) 6 (13.6) 0 (0.0) 0 (0.0) 0 (0.0)	157 (53.2) 72 (24.4) 52 (17.6) 11 (3.7) 1 (0.3) 2 (0.7)	79 (41.4) 54 (28.3) 32 (16.8) 14 (7.3) 1 (0.5) 11 (5.8)	
Length of stay (days)	7 [5–9]	7 [5–11]	7 [5–9]	8 [6–10]	0.002
In-hospital mortality	27 (5.1)	0 (0.0)	8 (2.7)	19 (9.9)	<0.001
**Chronic (home) HF medication**					
ARNI	20 (3.8)	1 (2.3)	15 (5.1)	4 (2.1)	0.207
ACEi/ARB	267 (50.4)	21 (47.7)	157 (53.2)	89 (46.6)	0.338
Beta-blocker	302 (57.0)	13 (29.5)	181 (61.4)	108 (56.5)	<0.001
MRA	114 (21.5)	3 (6.8)	61 (20.7)	50 (26.2)	0.016
SGLT2i	108 (20.4)	5 (11.4)	58 (19.7)	45 (23.6)	0.175
Furosemide	265 (50.0)	4 (9.1)	134 (45.4)	127 (66.5)	<0.001
Statin (any)	307 (57.9)	14 (31.8)	193 (65.4)	100 (52.4)	<0.001
**Discharge medication (survivors)**					
ARNI	55 (10.9)	18 (40.9)	31 (10.8)	6 (3.5)	<0.001
ACEi/ARB	197 (39.2)	20 (45.5)	137 (47.7)	40 (23.3)	<0.001
Beta-blocker	391 (77.7)	38 (86.4)	242 (84.3)	111 (64.5)	<0.001
MRA	293 (58.3)	35 (79.5)	169 (58.9)	89 (51.7)	0.004
SGLT2i	323 (64.2)	40 (90.9)	197 (68.6)	86 (50.0)	<0.001
Furosemide	446 (88.7)	32 (72.7)	254 (88.5)	160 (93.0)	<0.001
Statin (any)	331 (65.8)	29 (65.9)	210 (73.2)	92 (53.5)	<0.001
**Discharge medication, HFrEF subgroup (LVEF ≤ 40%)**					
ARNI	49/204 (24.0)	17/28 (60.7)	27/128 (21.1)	5/48 (10.4)	<0.001
Beta-blocker	183/204 (89.7)	26/28 (92.9)	120/128 (93.8)	37/48 (77.1)	0.004
MRA	145/204 (71.1)	27/28 (96.4)	90/128 (70.3)	28/48 (58.3)	0.002
SGLT2i	157/204 (77.0)	27/28 (96.4)	102/128 (79.7)	28/48 (58.3)	<0.001
**Follow-up outcomes**					
3-month mortality	62 (11.7)	1 (2.3)	19 (6.4)	42 (22.0)	<0.001
6-month mortality	95 (17.9)	1 (2.3)	35 (11.9)	59 (30.9)	<0.001
6-month ED visit (*n* = 422)	218 (51.7)	15 (34.9)	134 (52.3)	69 (56.1)	0.053
6-month AHF readmission (*n* = 422)	80 (19.0)	1 (2.3)	48 (18.8)	31 (25.2)	0.004
6-month terminal RRT	22 (4.2)	0 (0.0)	5 (1.7)	17 (8.9)	0.001
6-month combined endpoint	188 (35.5)	2 (4.5)	87 (29.5)	99 (51.8)	<0.001

**Table 5:** Twelve-month outcomes across frailty strata. Values are *n* (%). All-cause death, RRT, triple composite and in-hospital mortality are expressed over the full stratum; AHF rehospitalization among survivors and emergency-department visits are expressed among the 366 patients alive and free from terminal RRT at 12 months (denominators 42, 234 and 90) and are descriptive only. The cumulative incidence of first AHF rehospitalization is the Aalen–Johansen estimate in the full cohort with death and RRT as competing events (Gray’s test). Hazard ratio for all-cause death 3.29 (2.43–4.45) per stratum and 3.81 (2.40–6.04) for CFS ≥5 vs <5; cause-specific hazard ratio for first AHF rehospitalization 1.86 (1.42–2.45) and subdistribution hazard ratio 1.51 (1.19–1.92) per stratum. The lower rehospitalization count in the survivor-only descriptive analysis (94/366) reflects conditioning on 12-month survival and the checkpoint-based recording rule, whereas the competing-risks analysis used reconstructed first-event timing in the full cohort and therefore identified 151 first AHF rehospitalizations. AHF, acute heart failure; CFS, Clinical Frailty Scale; ED, emergency department; RRT, renal replacement therapy.

12-Month Outcome	CFS 1–3 (*n* = 44)	CFS 4–5 (*n* = 295)	CFS 6–9 (*n* = 191)	*p*
All-cause death	2 (4.5)	57 (19.3)	95 (49.7)	<0.001
AHF rehospitalization (12-month survivors)	2/42 (4.8)	63/234 (26.9)	29/90 (32.2)	0.003
AHF rehospitalization, cumulative incidence (full cohort)	4.5%	29.2%	33.0%	<0.001
Terminal renal replacement therapy	0 (0.0)	5 (1.7)	18 (9.4)	<0.001
Triple composite	4 (9.1)	124 (42.0)	130 (68.1)	<0.001
Emergency department visit	20/42 (47.6)	154/234 (65.8)	60/90 (66.7)	0.064
In-hospital mortality	0 (0.0)	8 (2.7)	19 (9.9)	<0.001

**Table 6:** Twelve-month all-cause mortality cross-classified by frailty (CFS ≥ 5) and NT-proBNP (median split, 5248 pg/mL). Analysis restricted to the 519 patients with an available admission NT-proBNP. CFS, Clinical Frailty Scale.

12-Month Mortality	Below-Median NT-proBNP	Above-Median NT-proBNP
**CFS < 5**	6.9% (*n* = 116)	22.0% (*n* = 59)
**Frail (CFS ≥ 5)**	24.3% (*n* = 144)	46.5% (*n* = 200)

**Table 7:** Incremental discrimination of frailty for 1-year mortality (*n* = 519, 149 deaths). CFS adjusted hazard ratio 2.54 (95% CI 1.78–3.62); per 1 standard deviation, 1.76 (1.42–2.18) for CFS and 1.71 (1.38–2.13) for log NT-proBNP. Apparent C-index confidence intervals by 300-sample bootstrap; optimism correction and the confidence interval for the ΔC-index by 500 bootstrap replications. CFS, Clinical Frailty Scale; eGFR, estimated glomerular filtration rate; LR, likelihood ratio; LVEF, left ventricular ejection fraction.

Model	Apparent C-Index (95% CI)	Optimism-Corrected C-Index	ΔC-Index (95% CI)	LR *p*
A: age + log NT-proBNP + eGFR + LVEF	0.701 (0.656–0.742)	0.694	—	—
B: model A + CFS	0.734 (0.694–0.778)	0.726	+0.033 (0.007–0.063)	<0.001

## Data Availability

The data supporting the findings of this study are not publicly available due to ethical considerations and patient confidentiality. Access to the dataset may be provided upon reasonable request to the corresponding author, subject to approval by the Bioethics and Ethics Committee of the Venizelio General Hospital of Heraklion.

## Supplementary Materials

The following supporting information can be downloaded at: https://www.mdpi.com/article/10.3390/medicina62101942/s1, Table S1: Chronic kidney disease (CKD) stages, KDIGO 2012 guidelines; Table S2: Worsening renal function (WRF) class; Table S3: Clinical phenotypes of acute heart failure at presentation, 2021 ESC guidelines; Table S4: Echocardiographic probability of pulmonary hypertension (PH), 2022 ESC/ERS guidelines; Methods S1: Classification of the first event under competing risks; Methods S2: Model diagnostics for the cardiorenal model with CFS; Methods S3: Proportional subdistribution hazards for the Fine–Gray model; Table S5: Tipping-point analysis for unobserved terminal readmissions; Table S6: Sensitivity to the event-classification rule; Figure S1: Kaplan–Meier all-cause survival over 12 months by frailty stratum; Figure S2: Calibration of the cardiorenal model with CFS; Table S7: Extended multivariable Cox model for 12-month all-cause mortality; Methods S4: Sensitivity to the competing-event specification.

## Abbreviations

The following abbreviations are used in this manuscript:

ACEi	Angiotensin-converting enzyme inhibitor
ACS	Acute coronary syndrome
AF	Atrial fibrillation
AHF	Acute heart failure
AI	Artificial intelligence
AKI	Acute kidney injury
ARB	Angiotensin receptor blocker
ARNI	Angiotensin receptor–neprilysin inhibitor
BP	Blood pressure
CABG	Coronary artery bypass grafting
CFS	Clinical Frailty Scale
CI	Confidence interval
CKD	Chronic kidney disease
CRP	C-reactive protein
EACVI	European Association of Cardiovascular Imaging
EASIX	Endothelial Activation and Stress Index
ED	Emergency department
eGFR	Estimated glomerular filtration rate
ERS	European Respiratory Society
ESC	European Society of Cardiology
HbA1c	Glycated hemoglobin
HDL	High-density lipoprotein
HF	Heart failure
HFrEF	Heart failure with reduced ejection fraction
ICD	Implantable cardioverter-defibrillator
IQR	Interquartile range
KDIGO	Kidney Disease: Improving Global Outcomes
LAVi	Left atrial volume index
LDL	Low-density lipoprotein
LR	Likelihood ratio
LV	Left ventricular
LVEF	Left ventricular ejection fraction
MRA	Mineralocorticoid receptor antagonist
NT-proBNP	N-terminal pro–B-type natriuretic peptide
NYHA	New York Heart Association
PASP	Pulmonary artery systolic pressure
PH	Pulmonary hypertension
RDW	Red-cell distribution width
RRT	Renal replacement therapy
RV	Right ventricular
SGLT2i	Sodium–glucose cotransporter-2 inhibitor
STEMI	ST-elevation myocardial infarction
TAPSE	Tricuspid annular plane systolic excursion
TIA	Transient ischemic attack
TSH	Thyroid-stimulating hormone
WBC	White blood cell count
WRF	Worsening renal function
