# Supplementary material for: Beyond Cardiorenal Risk Factors: The Prognostic Role of Frailty in Acute Heart Failure
Source: Medicina (Kaunas). 2026 Oct 8;62(10):1942. doi: 10.3390/medicina62101942 (PMC13707131; doi:10.3390/medicina62101942)
Supplement: Supplementary file 1 [file medicina-62-01942-s001.zip › FRAILTY_Supplementary.pdf]

Supplementary Material

Beyond Cardiorenal Risk Factors: The Prognostic Role of Frailty in Acute Heart Failure

Supplementary Data

Supplementary Table S1. Chronic kidney disease (CKD) stages, KDIGO 2012 guidelines. CKD was defined as eGFR <60 mL/min/1.73 m² persisting for ≥3 months. eGFR = estimated glomerular filtration rate.

CKD stage	eGFR (mL/min/1.73 m²)	Description
G1	≥90	Normal or high
G2	60–89	Mildly decreased
G3a	45–59	Mildly to moderately decreased
G3b	30–44	Moderately to severely decreased
G4	15–29	Severely decreased
G5	<15	Kidney failure

Supplementary Table S2. Worsening renal function (WRF) class. WRF was graded as an ordinal severity class from the ratio of peak in-hospital to baseline serum creatinine; the most severe grade (class 5) denotes initiation of in-hospital renal replacement therapy (RRT). Percentages are of the full cohort (n=530). RRT = renal replacement therapy.

Class	Peak/baseline serum creatinine ratio	n (%)
0	<1.25×	267 (50.4%)
1	1.25–1.50×	133 (25.1%)
2	1.51–2.00×	90 (17.0%)
3	2.01–3.00×	25 (4.7%)
4	>3.00×	2 (0.4%)
5	In-hospital RRT	13 (2.5%)

Supplementary Table S3. Clinical phenotypes of acute heart failure at presentation, 2021 ESC guidelines. Classified according to the 2021 ESC Heart Failure Guidelines. HF = heart failure; JVP = jugular venous pressure; RV = right ventricle; SBP = systolic blood pressure.

Phenotype	Key clinical features
Acute decompensated HF (ADHF)	Gradual worsening of pre-existing HF (dyspnea, edema); hemodynamically stable; no hypoperfusion. Most common presentation.
Acute pulmonary edema (APO)	Severe respiratory distress, orthopnea, low oxygen saturation, bilateral crackles; rapid onset; pulmonary congestion on imaging.
Isolated right heart failure	Predominant right-sided congestion (elevated JVP, peripheral edema, hepatomegaly) without significant left-sided congestion; reduced RV systolic function.
Cardiogenic shock	Hypotension (SBP <90 mmHg or vasopressors required) with end-organ hypoperfusion (oliguria, elevated lactate, cold peripheries, altered mentation) despite adequate filling.

Supplementary Table S4. Echocardiographic probability of pulmonary hypertension (PH), 2022 ESC/ERS guidelines. * Additional echocardiographic signs of PH from at least two different categories: RV/LV basal diameter ratio >1.0; flattening of the interventricular septum; RV outflow-tract acceleration time <105 ms and/or mid-systolic notching; early diastolic pulmonary regurgitation velocity >2.2 m/s; pulmonary artery diameter >25 mm; inferior vena cava diameter >21 mm with reduced inspiratory collapse; right atrial area >18 cm². TR = tricuspid regurgitation; RV = right ventricle; LV = left ventricle.

**TR peak velocity (m/s)**	**Additional PH signs ***	**PH probability**
≤2.8 or not measurable	No	Low
≤2.8 or not measurable	Yes	Intermediate
2.9–3.4	No	Intermediate
2.9–3.4	Yes	High
>3.4	Not required	High

Supplementary Methods S1. Classification of the first event under competing risks

Follow-up in this registry was administratively complete: every patient was observed until death or until 52 weeks, with no loss to follow-up. Vital status, AHF rehospitalization and renal replacement therapy (RRT) were recorded at prespecified 3-, 6- and 12-month checkpoints, whereas time to death and time to the first of death, AHF rehospitalization or RRT were recorded in weeks.

The checkpoint fields were completed hierarchically: when a terminal event and a non-fatal event occurred within the same checkpoint interval, only the terminal event was entered. As a consequence, the checkpoint fields systematically under-record rehospitalizations among patients who subsequently died, and an analysis restricted to survivors is conditioned on surviving to remain at risk. The recorded event times were therefore used to identify the first event and its type, according to the following prespecified rule:

(i) if the time to the composite endpoint preceded the time to death by at least one week, the patient was classified as having sustained a non-fatal event first — AHF rehospitalization, or RRT where an RRT episode but no rehospitalization was documented; (ii) if the two times coincided, the event was classified as death, because the endpoint of interest was non-fatal AHF rehospitalization and a readmission terminating in death within the same week represents a fatal decompensation rather than a non-fatal heart-failure event; (iii) patients without an event were censored at 52 weeks.

Under this rule, 151 patients sustained a first AHF rehospitalization, 93 died without a preceding rehospitalization, 14 had RRT as the first competing event, and 272 remained event-free or were administratively censored at 52 weeks (151 + 93 + 14 + 272 = 530). The 151 rehospitalizations include 3 patients whose only AHF rehospitalization was recorded at the final follow-up checkpoint, without an exact date; because the event coincided with the 52-week administrative cut-off, these events were assigned to the boundary week.

Supplementary Methods S2. Model diagnostics for the cardiorenal model with CFS

Proportional hazards (scaled Schoenfeld residuals): age p=0.27, log NT-proBNP p=0.27, admission eGFR p=0.44, LVEF p=0.18, CFS p=0.50. Departures from linearity (quadratic term added): age p=0.30, admission eGFR p=0.33, log NT-proBNP p=0.77. Neither assumption was violated.

Frailty modeled as a categorical rather than ordinal variable (reference CFS 1–3): CFS 4–5, adjusted hazard ratio 3.32 (95% CI 0.78–14.20); CFS 6–9, 8.25 (95% CI 1.85–36.74).

Standardized adjusted hazard ratios per one standard deviation: CFS 1.76 (95% CI 1.42–2.18); log NT-proBNP 1.71 (95% CI 1.38–2.13); age 1.08 (0.86–1.35); admission eGFR 0.89 (0.72–1.09); LVEF 0.97 (0.81–1.17).

Optimism correction by 500 bootstrap replications of the entire modeling procedure: apparent C-index 0.701 and 0.734, optimism 0.007 and 0.008, corrected 0.694 and 0.726; apparent ΔC-index 0.033 (paired bootstrap 95% CI 0.007–0.063); optimism-corrected ΔC-index 0.032. In a sensitivity model additionally adjusted for sex, de novo versus chronic HF, baseline NYHA class, admission sodium and albumin (513 complete cases, 147 deaths), frailty remained independently associated with mortality (adjusted hazard ratio 1.90 per stratum, 95% CI 1.25–2.90; likelihood-ratio χ^2=8.98, p=0.003) and continued to add discrimination (apparent C-index 0.744 with CFS versus 0.734 without CFS; bootstrap-corrected ΔC-index 0.010, 95% CI 0.001–0.027).

Supplementary Methods S3. Proportional subdistribution hazards for the Fine–Gray model

The proportional subdistribution hazards assumption for the Fine–Gray model of first AHF rehospitalization (frailty stratum as the covariate, death and RRT as competing events) was assessed with scaled Schoenfeld residuals on the subdistribution risk set. No violation was detected (χ^2=0.11, p=0.74). The subdistribution hazard ratio was likewise stable when estimated separately over the first and second halves of follow-up: 1.48 (95% CI 1.07–2.03) for weeks 0–26 and 1.62 (95% CI 0.97–2.71) for weeks 26–52, consistent with a constant subdistribution hazard over time. For completeness, the cause-specific Cox model for rehospitalization also satisfied the proportional-hazards assumption (χ^2=0.12, p=0.72).

Supplementary Table S5. Tipping-point analysis for unobserved terminal readmissions

Ninety-three deaths had a composite-endpoint time identical to the death time. Twenty-six of these occurred during the index hospitalization, in which a readmission is impossible by definition, leaving 67 post-discharge deaths in which a readmission occurring in the same week as death could not be excluded. Twenty-seven in-hospital deaths occurred in total (Table 5 of the main text); the remaining patient had in-hospital renal replacement therapy recorded as the first event and is therefore not among these 93 deaths. The table below reclassifies increasing proportions of these 67 as AHF rehospitalizations occurring immediately before death.

**Reclassified as AHF rehospitalization**	**Cumulative incidence, CFS 1–3 / 4–5 / 6–9 (%)**	**Cause-specific HR (95% CI)**	**Subdistribution HR (95% CI)**
None (primary analysis)	4.5 / 29.2 / 33.0	1.86 (1.42–2.45)	1.51 (1.19–1.92)
25% (17 of 67)	4.5 / 32.2 / 35.6	1.85 (1.42–2.40)	1.49 (1.16–1.93)
50% (34 of 67)	6.8 / 34.2 / 40.8	1.92 (1.49–2.46)	1.57 (1.23–2.00)
All (67 of 67)	9.1 / 38.0 / 62.5	2.11 (1.67–2.66)	1.81 (1.44–2.27)

The larger increase across frailty strata for mortality than for documented non-fatal rehospitalization was preserved in the primary analysis and in the partial-reclassification scenarios (25% and 50% reclassified); only under the deliberately extreme assumption that every such post-discharge death was preceded by a terminal AHF readmission did the rehospitalization gradient (9.1%→62.5%) exceed the mortality gradient (4.5%→49.7%). The hazard ratio for all-cause death was 3.29 (95% CI 2.43–4.45) per frailty stratum in every scenario; hazard ratios for the different endpoints are reported separately and should not be directly compared because they represent different estimands.

Supplementary Table S6. Sensitivity to the event-classification rule

An alternative, more conservative rule counts an AHF rehospitalization only where it is explicitly documented in a checkpoint field, disregarding the recorded event times. Because 20 of the 23 discrepant events fell in intervals for which no checkpoint was ever completed — the patient having died before the next scheduled assessment — this rule reintroduces the survivorship bias that the competing-risks analysis is intended to remove, and is presented only to display the range of the estimate. The remaining 3 were the boundary-week rehospitalizations described in Supplementary Methods S1; these are recorded in the 12-month checkpoint field but, lacking an exact date, coincide with the 52-week administrative cut-off and are therefore not counted as within-follow-up events under the checkpoint-only rule.

**Classification rule**	**Events (n)**	**Cumulative incidence, CFS 1–3 / 4–5 / 6–9 (%)**	**Cause-specific HR (95% CI)**
Event-time based (primary)	151	4.5 / 29.2 / 33.0	1.86 (1.42–2.45)
Checkpoint fields only	128	4.5 / 27.5 / 23.6	1.53 (1.14–2.04)

Supplementary Figure S1. Kaplan–Meier all-cause survival over 12 months by frailty stratum (log-rank $p<0.001$). CFS, Clinical Frailty Scale.

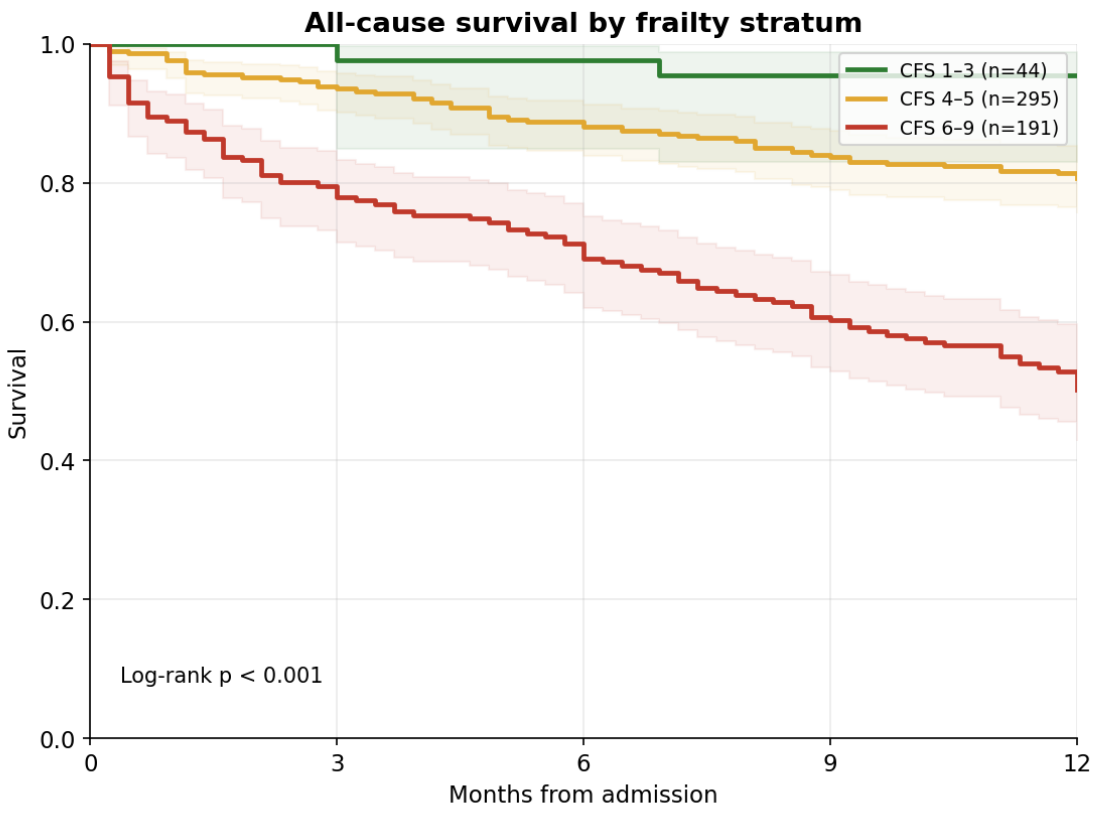

Supplementary Figure S2. Calibration of the cardiorenal model with CFS

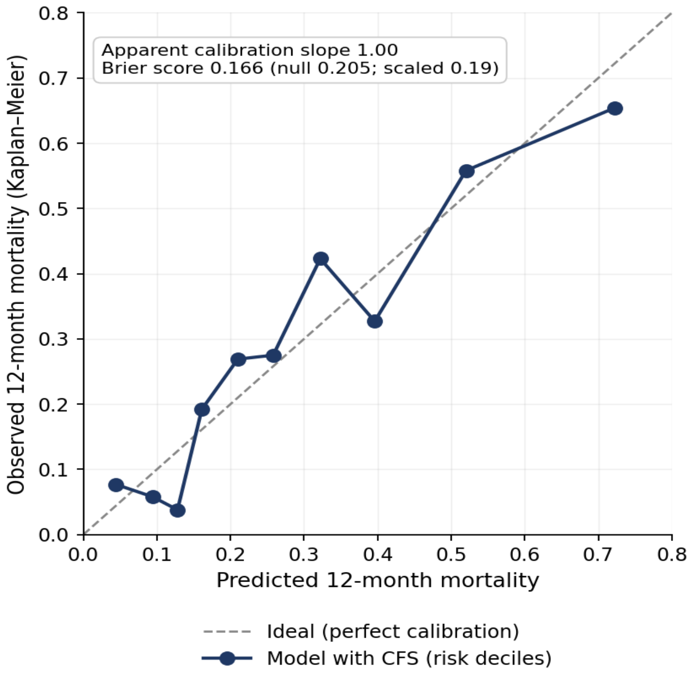

Calibration of predicted versus observed 12-month mortality for the model including CFS (age, log NT-proBNP, admission eGFR, LVEF and CFS), by deciles of predicted risk. Observed mortality is the Kaplan–Meier estimate within each decile. The calibration slope was 1.00 and the Brier score 0.166 (null model 0.205; scaled Brier 0.19). This figure presents apparent calibration within the development cohort; optimism-corrected calibration was not derived.

Supplementary Table S7. Extended multivariable Cox model for 12-month all-cause mortality

Covariate	Adjusted HR (95% CI)	p
Age (per year)	1.00 (0.98–1.03)	0.75
Male sex	1.44 (1.01–2.06)	0.046
De novo vs chronic HF	0.63 (0.37–1.06)	0.082
Baseline NYHA class	1.40 (0.97–2.03)	0.074
log NT-proBNP	1.73 (1.37–2.19)	<0.001
Admission eGFR	1.00 (0.99–1.01)	0.38
Admission sodium	0.99 (0.95–1.03)	0.50
Albumin	0.72 (0.46–1.14)	0.17
LVEF	1.00 (0.99–1.02)	0.55
Frailty (per CFS stratum)	1.90 (1.25–2.90)	0.003

Complete-case analysis (n=513, 147 deaths); NT-proBNP was missing in 11 and albumin in 6 patients, analyzed as complete cases. Harrell C-index 0.734 without CFS and 0.744 with CFS; likelihood-ratio χ^2=8.98 (p=0.003) for the addition of CFS; bootstrap-corrected ΔC-index 0.010 (95% CI 0.001–0.027, 400 replications). HR, hazard ratio; CFS, Clinical Frailty Scale; HF, heart failure; NYHA, New York Heart Association; eGFR, estimated glomerular filtration rate; LVEF, left ventricular ejection fraction.

Supplementary Methods S4. Sensitivity to the competing-event specification

To confirm that the choice of competing events did not drive the rehospitalization findings, the analysis was repeated with death as the sole competing event, censoring patients at the initiation of terminal renal replacement therapy rather than treating it as a competing event. The 12-month cumulative incidence of first AHF rehospitalization was essentially unchanged (4.5%, 29.3% and 33.7% across CFS strata 1–3, 4–5 and 6–9; Gray's test $p<0.001$), the cause-specific hazard ratio was essentially unchanged (1.84, 95% CI 1.40–2.42), and the subdistribution hazard ratio was marginally higher (1.57, 95% CI 1.23–2.00). The findings were therefore robust to the treatment of renal replacement therapy.
